# Cysteine-specific ubiquitination protects the peroxisomal import receptor Pex5p against proteasomal degradation

**DOI:** 10.1042/BSR20150103

**Published:** 2015-06-22

**Authors:** Benjamin Schwartzkopff, Harald W. Platta, Sohel Hasan, Wolfgang Girzalsky, Ralf Erdmann

**Affiliations:** * Institut für Biochemie und Pathobiochemie, Abteilung für Systembiochemie, Medizinische Fakultät der Ruhr-Universität Bochum, D-44780 Bochum, Germany; † Biochemie Intrazellulärer Transportprozesse, Medizinische Fakultät der Ruhr-Universität Bochum, D-44780 Bochum, Germany

**Keywords:** peroxisome biogenesis, Pex5p, protein import, type 1 peroxisomal targeting signal (PTS1)-receptor, ubiquitination

## Abstract

Peroxisomal matrix protein import is mediated by dynamic import receptors, which cycle between the peroxisomal membrane and the cytosol. Proteins with a type 1 peroxisomal targeting signal (PTS1) are bound by the import receptor Pex5p in the cytosol and guided to the peroxisomal membrane. After cargo translocation into the peroxisomal matrix, the receptor is released from the membrane back to the cytosol in an ATP-dependent manner by the AAA-type ATPases Pex1p and Pex6p. These mechanoenzymes recognize ubiquitinated Pex5p-species as substrates for membrane extraction. The PTS1-receptor is either polyubiquitinated via peptide bonds at two certain lysines and results in proteasomal degradation or monoubiquitinated via a thioester-bond at a conserved cysteine, which enables the recycling of Pex5p and further rounds of matrix protein import. To investigate the physiological relevance of the conserved N-terminal cysteine of Pex5p, the known target amino acids for ubiquitination were substituted by site-directed mutagenesis. In contrast with Pex5p_C6A_, Pex5p_C6K_ turned out to be functional in PTS1 import and utilization of oleic acid, independent of the lysines at position 18 and 24. In contrast with wild-type Pex5p, Pex5p_C6K_ displays an ubiquitination pattern, similar to the polyubiquitination pattern of Pex4p or Pex22p mutant strains. Moreover, Pex5p_C6K_ displays a significantly reduced steady-state level when the deubiquitinating enzyme Ubp15p is missing. Thus, our results indicate that not the cysteine residue but the position of ubiquitination is important for Pex5p function. The presence of the cysteine prevents polyubiquitination and rapid degradation of Pex5p.

## Abstract (precis)

Peroxisomal import receptors cycle between the peroxisomal membrane and the cytosol. A monoubiquitinated cysteine is required for efficient recycling of the peroxisomal import receptor Pex5p and prevents the protein from polyubiquitination, which leads to a rapid degradation of the protein.

## INTRODUCTION

Peroxisomes are ubiquitous organelles, which carry out a wide variety of metabolic processes in eukaryotic organisms [1]. Therefore, the dysfunction of these organelles in humans results in severe peroxisomal disorders [2–4]. The functionality of these organelles is governed by dynamically operating import machineries for peroxisomal membrane and matrix proteins [1,5]. Peroxisomal matrix proteins are without exception nuclear encoded, synthesized on free ribosomes and subsequently recognized in the cytosol by specific soluble receptors [6,7]. To this end, cargo proteins are equipped with a targeting sequence, either a C-terminal PTS1 (peroxisomal targeting signal type 1) or an N-terminal PTS2, which are recognized and bound by the import receptor Pex5p or Pex7p respectively [8]. Subsequent to the assembly in the cytosol, the receptor ferries its cargo to the peroxisomal membrane, where it associates with the docking complex and then becomes part of a dynamic translocation pore [9]. The molecular mechanism underlying the translocation and release of the cargo remains elusive. Finally, the cargo-free receptors are exported back to the cytosol in an ATP-dependent manner. This step is catalysed by the AAA (ATPases associated with various cellular activities) peroxins Pex1p and Pex6p, which are supposed to function as mechanoenzymes by pulling the receptor out of the membrane [10,11]. Ubiquitination of the receptor serves as a signal for this process [12–14].

Two kinds of ubiquitination of the Pex5p receptor have been reported, namely mono- and polyubiquitination. Monoubiquitination primes the receptor for its export back to the cytosol to allow a new round of matrix protein import [12,14,15]. Pex5p monoubiquitination occurs at a conserved cysteine residue near the N-terminus of Pex5p (Cys^6^ in *Saccharomyces cerevisiae*) and is facilitated by the E2 ubiquitin-conjugation enzyme Pex4p in cooperation with the RING (really interesting new gene)-type E3 ligase Pex12p. Pex12p is part of the peroxisome membrane-associated RING sub-complex of E3 ligases (Pex2p, Pex10p and Pex12p) [12,15–17]. Alternatively, in cells lacking a component of the receptor recycling machinery, the receptor is subjected to polyubiquitination, which occurs on conserved lysines near the N-terminus of Pex5p (Lys^18^/Lys^24^ in *S. cerevisiae*) and leads to receptor degradation [18–21]. In *S. cerevisiae*, polyubiquitination of Pex5p depends on the cytosolic E2 enzyme Ubc4p (ubiquitin conjugating enzyme) and the E3 ligase Pex10p [22] or Pex2p with the assistance of Pex10p [17,23].

Usually, Gly^76^ of the ubiquitin is attached to a ε-NH_2_ group of a lysine residue of the target protein by formation of an isopeptide bond or in some cases to the α-NH_2_ group of the extreme N-terminus. As an exception to the rule, also cysteine residues can be targeted by ubiquitination machineries. Apart from the PTS-receptors Pex5p, Pex18p and Pex20p [14,16,21,24,25], only a few cases of ester-based ubiquitination substrates are known, like e.g. tBid (truncated BH3 interacting domain death agonist), MHC-I (major histocompatibility complex I) heavy chain or TCRα (T cell receptor alpha chain) [26]. In these cases, the ubiquitination of substrates via esterification serves to provide more target residues in order to enhance degradation. In contrast, the esterification of the PTS1-receptor seems to have a physiologically relevant mechanistic function, because it is required for the recycling and reuse of Pex5p for further rounds of matrix protein import.

In order to elucidate the physiological relevance of the conserved N-terminal cysteine of PTS1-receptor, Pex5p-variants were generated containing substitution of the corresponding cysteine and other known target amino acids for ubiquitination by lysine, alanine or arginine. Our data show that Pex5p_C6K_ is functional in PTS1 import. However, instead of the typical monoubiquitination, the Pex5p_C6K_ is polyubiquitinated. Moreover, Pex5p_C6K_ displays significantly reduced steady state level when the deubiquitinating enzyme Ubp15p (ubiquitin-specific protease) is missing. Thus, our results indicate that not the cysteine residue but the position of ubiquitination is important for the general function of Pex5p in peroxisome biogenesis. However, the cysteine is required for efficient recycling of Pex5p and prevents the protein from polyubiquitination, which leads to a rapid degradation of the protein.

## EXPERIMENTAL

### Yeast strains and culture conditions

Wild-type *Saccharomyces cerevisiae* strain UTL-7A (MATa, ura3-52, trp1, leu2-3/112) [27] was the isogenic source for the generation of used *pex1*Δ [18], *pex4Δ* [28], *pex5Δ *[29], *pex4Δ pex5Δ* [12], *ubp15*Δ [30], as well as *ubp15*Δ*pex5*Δ strains (present study). Deletion strains were generated by the ‘short flanking homology’ method as described before [31]. Yeast media have been described previously [27]. For induction of the *CUP1* promoter, CuSO_4_ was added according to [32]. When the proteasome inhibitor MG132 was used, the medium was supplemented with 0.1% (w/v) proline and 0.003% (w/v) SDS to allow the uptake of the inhibitor according to [33].

### Yeast cell extracts

Yeast cells were grown on 0.3% YEPD medium to late exponential phase and subsequently for 15 h in YNOD [0.1% (w/v) dextrose, 0.1% (w/v) oleic acid, 0.05% (v/v) Tween 40, 0.1% (w/v) yeast extract and 0.67% (w/v) yeast nitrogen base]. Cells were harvested and aliquots of 30 mg of cells were resuspended in 300 μl of potassium phosphate buffer (pH 7.4) containing 20% trichloroacetic acid. The samples were frozen at −80**°**C for at least 30 min. Samples were sedimented, washed twice with ice-cold 80% acetone and resuspended in 80 μl of 1% (w/v)

SDS/0.1M NaOH and 20 μl of SDS loading buffer [5% (w/v) 2-mercaptoethanol, 15% (v/v) glycerol and 0.01% (w/v) Bromophenol Blue].

### Plasmids

Used plasmids and oligonucleotides are listed in Tables 1 and 2 respectively. Pex5p was expressed from a low-copy vector under the control of its own promoter (pHP17-PEX5) [12]. Point mutations in *PEX5* were introduced using overlap extension PCR leading to the lysine to arginine, cysteine to lysine as well as cysteine to alanine mutant collection of *PEX5*.

**Table 1:** Plasmids used

Plasmid	Description	Source or reference	Oligonucleotides
pHP17-PEX5	Pex5p[WT]	[73]	–
pRS416-PEX5-C6A	Pex5p[C6A]	present study	RE3318/RE3319
pRS416-PEX5-C6K	Pex5p[C6K]	present study	RE2600/RE2601
pHP18-PEX5	Pex5p[K18R/K24R]	[73]	–
pRS416-PEX5-C6K-K18R/K24R	Pex5p[C6K/K18R/K24R]	present study	–
pRS416-PEX5-C6A-K18R/K24R	Pex5p[C6A/K18R/K24R]	present study	*–*
YEP96	CUP1-Ubiquitin	[74]	*–*
YEP105	CUP1-*myc*Ubiquitin	[42]	–
pUG34DsRed.SKL	DsRed-SKL	[75]	–
pRS416-PEX5-K18C	Pex5p[K18C]	present study	RE3838/RE3839
pRS416-PEX5-C6A-K18C	Pex5p[C6A/K18C]	present study	RE3838/RE3839

**Table 2:** Oligonucleotides used

Oligonucleotide	Sequence
RE3318	5′-GACCATGGACGTAGGAAGTGCCTCAGTGGGAAATAATCCG-3′
RE3319	5′-CGGATTATTTCCCACTGAGGCACTTCCTACGTCCATGGTC-3′
RE2600	5′-ATGGACGTAGGAAGTAAATCAGTGGGAAATAATCCGCTTGCGC-3′
RE2601	5′-GCGCAAGCGGATTATTTCCCACTGATTTACTTCCTACGTCCAT-3′
RE3838	5′-AATCCGCTTGCGCAGTTGCACTGCCATACTCAGCAGAACAAATCG-3′
RE3839	5′-CGATTTGTTCTGCTGAGTATGGCAGTGCAACTGCGCAAGCGGATT-3′

### Microscopy

Fluorescence microscopy of live cells for DsRed (*Discosoma* sp. RFP) expression and localization was performed with a Zeiss Axioplan microscope and AxioVision 4.1 software (Zeiss). Before inspection, cells were grown for 2 days on solid minimal medium containing oleic acid as a sole carbon source. Both fluorescence and optical photographs were taken by using the connected hardware in combination with the Spot RT software version 3.1 (Diagnostics Instruments). Adjustments of contrast and brightness were carried out with Adobe Photoshop software version 13.0 and characteristic cells were cut-out and copied to Adobe Illustrator version CS6.

### Miscellaneous

Immunoblot analyses were performed according to standard protocols [34]. Immunoblots were incubated with polyclonal rabbit antibodies raised against Pex5p [35] and mitochondrial porin [36]. Primary antibody was detected with an IRDye 800CW goat anti-rabbit IgG secondary antibody (LI-COR Bioscience) followed by a detection using the ‘Infrarot Imaging System’ (LI-COR Bioscience).

## RESULTS

### Functional expression of Pex5p_C6K_

On the basis of functional and structural data, the PTS1-receptor Pex5p comprises two separate parts (Figure 1A). Whereas the C-terminal TPR (tetratricopeptide repeat) domain is highly conserved and facilitates binding to PTS1-cargo proteins, the N-terminal domain hosts most of the interaction sites for other peroxins but reveals poor conservation [37]. Interestingly, the extreme N-terminal part contains conserved amino acid residues serving as targets for ubiquitination. The conserved lysines at position 18 and 24 of yeast Pex5p are required for polyubiquitination and subsequent degradation [12,19]. In contrast and as an exception to the rule, a conserved cysteine at position 6 undergoes monoubiquitination [16], which is required for the Pex1p/Pex6p-dependent release of the receptor from the peroxisomal membrane to the cytosol [16,38]. In order to gain more insight into functional relevance of this atypical linkage of ubiquitin to the substrate protein via a thioester bond to a cysteine, we substituted this conserved amino acid by site directed mutagenesis to alanine or lysine.

**Figure 1:**
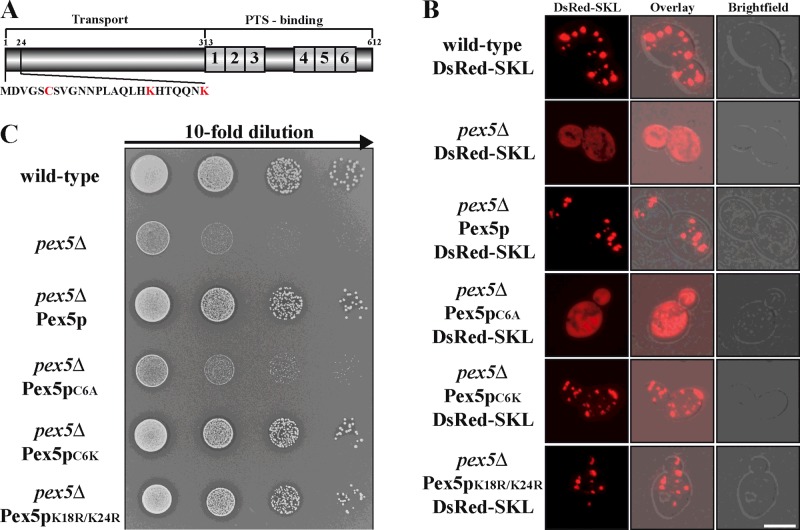
Pex5p_C6K_ can complement a *pex5*Δ strain (**A**) Scheme of *S. cerevisiae* Pex5p. Pex5p contains an N-terminal domain (1–312) with interaction sites required for protein transport and a C-terminal domain (313–612) with six TPR domains (1–6) responsible for the binding of PTS1-proteins. The extreme N-terminus contains the target amino acids for mono- (Cys^6^) and polyubiquitination (Lys^18^ and Lys^24^) highlighted in red. (**B**) Indicated strains were spotted as a series of 10-fold dilutions on a medium with oleic acid as sole carbon source and incubated for 4 days at 30°C. Mutant *pex5*Δ and *pex5*Δ expressing Pex5p_C6A_ were unable to grow on oleic acid medium. In contrast, the mutant growth phenotype was complemented upon expression of Pex5p, Pex5p_K18R/K24R_ and Pex5p_C6K_, which display a growth behaviour similar to the wild-type. (**C**) Oleic acid-induced indicated strains were analysed for the subcellular localization of the transformed PTS1-marker DsRed-SKL by fluorescence microscopy. Mutant *pex5*Δ and *pex5*Δ expressing Pex5p_C6A_ exhibited an overall cellular fluorescence, indicative for a mislocalization of the peroxisomal marker protein as consequence of a peroxisomal import defect. Mutant cells expressing wild-type Pex5p or mutant Pex5p_K18R/K24R_ or Pex5p_C6K_ imported PTS1-proteins properly as indicated by the punctate fluorescence pattern. Scale bar=5 μm

Wild-type and *pex5Δ* mutant strain and transformants were tested for their ability to import the fluorescence marker DsRed fused to type I targeting signal Ser-Lys-Leu (SKL). In wild-type cells, DsRed-SKL appeared in punctate structures indicative for a peroxisomal localization (Figure 1B). In contrast, the synthetic peroxisomal matrix protein mislocalized to the cytosol in *pex5Δ* cells, which is in line with the specific import defect of this mutant [39]. This mutant phenotype was functionally complemented upon expression of wild-type Pex5p, whereas Pex5p_C6A_ failed to restore the import defect of *pex5Δ* cells. This finding is in line with previous reports, which demonstrated that the corresponding Pex5p-version of yeast and mammals is not monoubiquitinated anymore and therefore blocked for export from the peroxisomal membrane back to the cytosol [16,40]. In contrast, Pex5p_K18R/K24R_, which lacks the target residues for polyubiquitination, was still functional (Figure 1B; [12]). Remarkably, when the cysteine at position 6 was replaced by lysine (Pex5p_C6K_), peroxisomal import of the PTS1-marker protein DsRed-SKL was not affected (Figure 1B). The PTS1-receptor is not only required for the peroxisomal import of typical PTS1-proteins but also for non-PTS proteins like acyl-CoA oxidase, which bind to the receptor on its N-terminal half [5,41]. We extended our functional assay and monitored the strains for growth on plates containing oleic acid as the sole carbon source, which supports cell growth only if peroxisomal β-oxidation is functional. The *pex5Δ-*strain was unable to grow on this medium in line with previous findings [39] and typical for peroxisomal mutant strains of *S. cerevisiae* [27]. Whereas Pex5p_C6A_ failed to restore the growth defect of *pex5Δ*, the mutant strains expressing wild-type Pex5p, Pex5p_K18R/K24R_ or Pex5p_C6K_ grew at a rate indistinguishable from the wild-type strain (Figure 1C). Accordingly, we conclude that Pex5p_C6K_ can fully restore the function of native Pex5p in peroxisomal protein import.

### Position-dependent ubiquitination is required for Pex5p function

Inspired by the finding that a lysine residue on position 6 can functionally replace the conserved cysteine residue of Pex5p, we investigated the effect of shifting the conserved cysteine to position 18, thereby replacing a lysine, which is the usual target for polyubiquitination. We also combined this Pex5p-variant with the C6A substitution. The single substitution as well as combinations displayed similar steady-state levels (Figure 2A), indicative for comparable expression and stability. We monitored the capability of the Pex5p-variants to functionally complement the growth defect of the *pex5*Δ-strain on oleic acid medium and found that the substitution of Lys^18^ by cysteine has no significant effect on Pex5p function as long as the cysteine at position 6 is present (Figure 2B). However, the Pex5p-variant Pex5p_C6A/K18C_ could not restore the ability of the *pex5*Δ-strain to grow on oleic acid medium, indicating that the presence of cysteine at position 18 could not compensate for the loss of the conserved cysteine at position 6, (Figure 2B). We conclude that the position of the target residue for ubiquitination is crucial, regardless of whether a cysteine or lysine residue is present.

**Figure 2:**
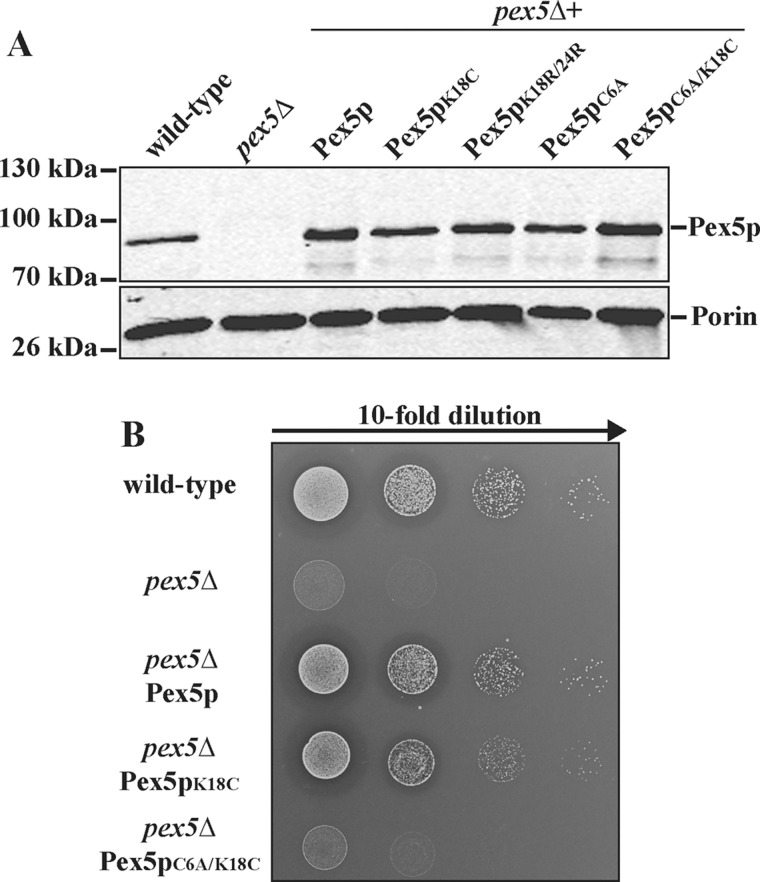
Position-dependent ubiquitination is required for Pex5p function Mutant *pex5*Δ cells were transformed with plasmids expressing Pex5p or indicated variants. (**A**) Immunoblot analysis of equal amounts of whole-cell trichloroacetic acid lysates of indicated strains with antibodies against Pex5p. Detection of mitochondrial porin served as loading control. (**B**) Indicated strains were spotted as a series of 10-fold dilutions on a medium with oleic acid as sole carbon source and incubated for 4 days at 30°C. Growth of mutant *pex5*Δ cells expressing Pex5p or Pex5p_K18C_ was indistinguishable from the wild-type, whereas the mutant *pex5*Δ cells expressing Pex5p_C6A/K18C_ display no growth on this carbon source.

### Pex5p_C6K_ is artificially polyubiquitinated independent of Lys^18^ and Lys^24^

Next, we analysed the consequence of the functional exchange of the conserved cysteine by lysine to the ubiquitination status of Pex5p. Whole-cell lysates of oleic acid-induced wild-type cells or *pex5*Δ cells expressing wild-type or mutant variants of Pex5p were prepared and subjected to immunoblot analysis. The steady-state level of plasmid-encoded wild-type and Pex5p-variants was similar (Figure 3A). The plasmid-encoded proteins showed an approximately four-fold higher steady-state level than endogenously expressed Pex5p (Figure 3A). Interestingly, Pex5p_C6K_ displayed high molecular mass species, which were not seen for wild-type Pex5p. We suspected that these Pex5p-species represent ubiquitinated forms of the PTS1-receptor, most probably Pex5p polyubiquitination. As Pex5p polyubiquitination is known to depend on the conserved lysine residues at position 18 and 24 [12], we additionally substituted these amino acid residues by arginine. The resulting Pex5p_K18R/K24R_ remained unmodified even when combined with C6A substitution (Figure 3A). However, combination with the C6K mutation still resulted in the modification of the receptor as observed for Pex5p_C6K_. Thus, the Pex5p modification is independent of the Lys^18^ and Lys^24^. To analyse whether this modification indeed represents ubiquitination, we analysed *pex5*Δ Pex5p_C6K/K18R/K24R_ cells additionally expressing either the wild-type Ub (ubiquitin) or the *myc*Ub fusion [42]. The *myc*Ub variant is about 1.5 kDa larger than wild-type Ub and leads to a shift of modified proteins [42]. Whole-cell lysates of the transformants were prepared and equal amounts of protein from each strain were subjected to immunoblot analysis. Samples were probed with anti-Pex5p antibodies and slower migrating Pex5p species appeared (Figure 3B). Comparison of samples from *pex5*Δ Pex5p_C6K/K18R/K24R_ expressing either Ub or *myc*Ub showed that the putative ubiquitinated species of Pex5p were replaced by slightly larger new bands, clearly demonstrating that Pex5p_C6K/K18R/K24R_ is polyubiquitinated at Cys^6^ *in vivo* under wild-type conditions.

**Figure 3:**
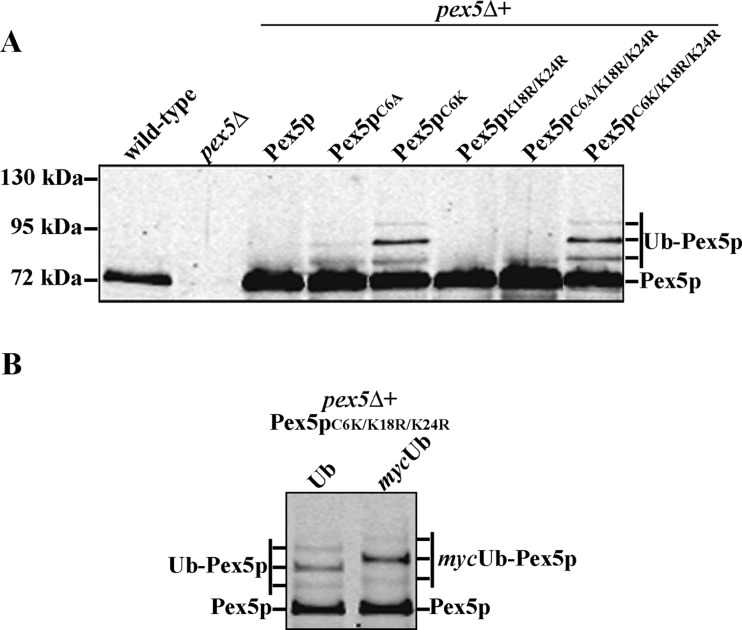
Pex5p_C6K_ is artificially polyubiquitinated independent of K18 and K24 Mutant *pex5*Δ cells were transformed with plasmids expressing Pex5p or indicated variants. (**A**) Immunoblot analysis of equal amounts of whole-cell trichloroacetic acid lysates of indicated strains with Pex5p-specific antibodies. In contrast with the wild-type, *pex5*Δ expressing Pex5p_C6A_ shows up to three additional αPex5p reactive bands. The third and especially the second band are more pronounced in case of Pex5p_C6K_ and Pex5p_C6K/K18R/K24R_. (**B**) The *pex5*Δ strain expressing Pex5p_C6K/K18R/K24R_ was additionally transformed with a plasmid either encoding ubiquitin (Ub) or *myc*-tagged ubiquitin (*myc*Ub). The observed shift to a higher molecular mass upon *myc*Ub expression proved that the additional αPex5p-reactive bands represent ubiquitinated Pex5p.

### Deletion of *PEX4* alters the Pex5p_C6K_ modification pattern

Polyubiquitination of Pex5p was initially observed in strains affected in components of the peroxisomal AAA-(Pex1p, Pex6p, Pex15p) or the Pex4p–Pex22p-complex [18,19]. Pex5p ubiquitination was also seen in strains with unaffected peroxisomal biogenesis but reduced proteasomal function, indicating that Pex5p-polyubiquitination also occurs under wild-type conditions [18]. Driven by the finding that Pex5p_C6K/K18R/K24R_ is polyubiquitinated, we compared the Ub-pattern with those observed in a strain affected in *PEX1* or *PEX4*. In contrast with *pex1*Δ strain in which Pex5p displays four Ub-species, Pex5p_C6K/K18R/K24R_ pattern was similar to those present in *pex4*Δ cells, although with different intensity of the appearing bands (Figure 4A). In *pex4*Δ the fastest migrating Ub-Pex5p species was most prominent, likely to represent monoubiquitinated Pex5p. In contrast, the second Ub-species was most abundant of Pex5p_C6K/K18R/K24R_, most probably representing di-ubiquitinated Pex5p, which might indicate a role of Pex4p in this modification. Next we analysed the Ub-status of Pex5p-variants in *pex5*Δ mutants and in *pex5*Δ*pex4*Δ double mutants. Pex5p_C6A_ and Pex5p_K18R/K24R_ behaved like wild-type Pex5p and thus displayed no significant polyubiquitination pattern in *pex5*Δ cells. Pex5p_C6A_ exhibited the typical *pex4*Δ-pattern when expressed in *pex5*Δ*pex4*Δ cells (Figure 4B). In contrast, Pex5p_C6K_ as well as Pex5p_C6K/K18R/K24R_ displayed the pattern of three Ub-species from which the intensity of the dominant middle band was shifted to the lowest band and thus lead to a typical *pex4*Δ pattern. From this, we conclude that Pex4p is involved, either directly or indirectly in the ubiquitination of Pex5p_C6K_.

**Figure 4:**
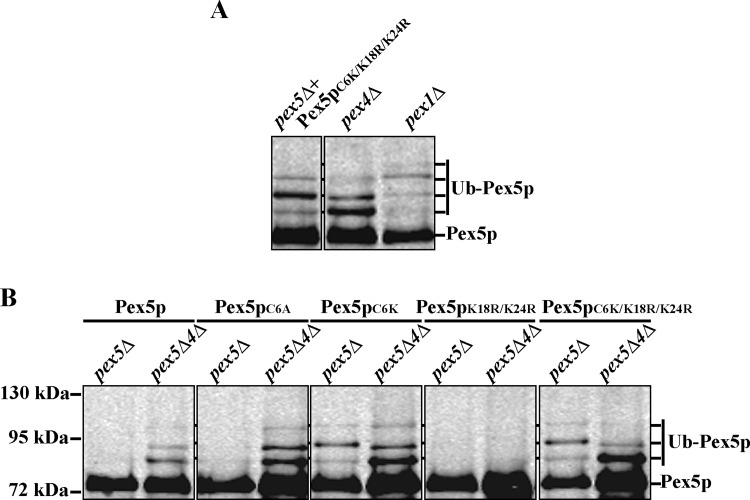
Ubiquitination profile of Pex5p_C6K/K18R/K24R_ is altered by Pex4p Immunoblot analysis of whole-cell trichloroacetic acid lysates of indicated strains with specific Pex5p antibodies. (**A**) Mutant *pex5*Δ cells were transformed with plasmids expressing Pex5p_C6K/K18R/K24R_. The ubiquitination pattern of the Pex5p-variant expressed in mutant *pex5*Δ cells is different from the Pex5p-modification pattern of *pex1*Δ cells, but resembles the pattern for Pex5p of a *pex4*Δ strain, although with shifted intensities. (**B**) Ubiquitination pattern of indicated Pex5p variants expressed in mutant *pex5*Δ and in double-mutant *pex5*Δ*pex4*Δ cells. The *pex4*Δ-ubiquitination pattern is observed for Pex5p variants when Pex4p is absent, but is more pronounced than in the wild-type. In the Pex5pC6K variants, the second Ub-Pex5p band from below shows a small shift to a lower molecular mass.

### Artificial ubiquitination of Pex5p_C6K_ is linked to higher turnover rate

Polyubiquitinated proteins are in general designated for proteasomal degradation and usually exhibit a high turnover rate, whereas ubiquitin itself is quite stable [43,44]. This is achieved by specific deubiquitinating enzymes, which remove the ubiquitin moieties prior to the actual degradation process [45–47]. Previously, the deubiquitinating enzyme Ubp15p (or ubiquitin-specific protease 9X in mammals, [48]) was identified as part of the cytosolic Pex1p–Pex6p complex in *S. cerevisiae* [30]. Ubp15p associates with the AAA-complex by an interaction with Pex6p and the recombinant protein is capable to deubiquitinate Pex5p at isolated membranes. In order to analyse whether the observed higher ubiquitination rate leads to a fast degradation of Pex5p_C6K_, we analysed the steady-state level of the PTS1-receptor in wild-type cells and in cells lacking Ubp15p. As judged by immunoblot analysis, the Pex5p_C6K_ level was already slightly reduced in comparison with native Pex5p when expressed in *pex5*Δ cells (Figure 5). The same was observed for Pex5p_C6K/K18R/K24R_. However, the effect was more significant in a strain lacking Ubp15p. Here, the amount of Pex5p_C6K_ as well as the Pex5p_C6K/K18R/K24R_ was significantly reduced. Quantification of protein amount by densitometry is shown in Figure 5(B). The data suggest that the reduced level of Pex5p_C6K_ variants is the consequence of ubiquitin-triggered increased degradation of Pex5p by the 26S proteasome.

**Figure 5:**
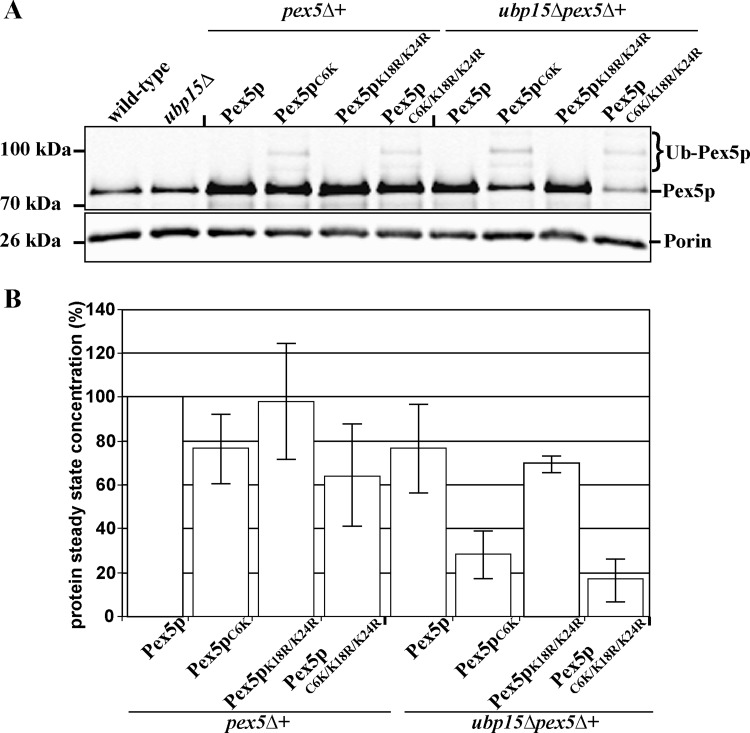
Artificial ubiquitination of Pex5p_C6K_ is linked to higher turnover rate Mutant *pex5*Δ cells or *ubp15*Δ*pex5*Δ cells were transformed with plasmids expressing Pex5p or indicated variants. (**A**) Whole-cell lysates of oleic acid-induced strains as indicated were subjected to immunoblot analysis with Pex5p-specific antibodies. Mitochondrial Porin served as control for equal loading. (**B**) Signal intensities of Pex5p of three independent experiments were estimated by densitometry. Obtained signal intensities were normalized to signal from plasmid-encoded wild-type Pex5p expressed in *pex5*Δ cells. The steady-state concentration of Pex5p_C6K_ and especially Pex5p_C6K/K18R/K24R_ was drastically reduced in the absence of Ubp15p. Error bars=S.E.M.

To test this assumption, we analysed whether Pex5p variants can be stabilized by inhibition of proteasomal degradation. To this end, oleic acid-induced *pex5Δ* or *pex5Δubp15Δ* cells expressing wild-type Pex5p, Pex5p_C6K_ or Pex5p_C6K/K18R/K24R_ were shifted to fresh medium with and without the proteasome inhibitor MG132. After additional growth for 4 h to allow uptake and functional development of the inhibitor, whole-cell lysates were prepared and analysed for the presence of Pex5p and its variants. As judged by immunoblot analysis and signal quantification, the level of wild-type Pex5p increased only slightly upon proteasomal inhibition (Figure 6). However, when analysed in the *pex5Δubp15Δ* strain, the Pex5p_C6K_ variant and especially the Pex5p_C6K/K18R/K24R_ form was decreased in the absence of MG132, but proteasomal inhibition did result in a significant stabilization of the protein. Under these conditions about four-fold higher protein level was detected for Pex5p_C6K/K18R/K24R_. The data show that substitution of the conserved cysteine residue of the Pex5p N-terminus by lysine leads to artificial polyubiquitination followed by a higher degradation rate of the receptor by the 26S proteasome.

**Figure 6:**
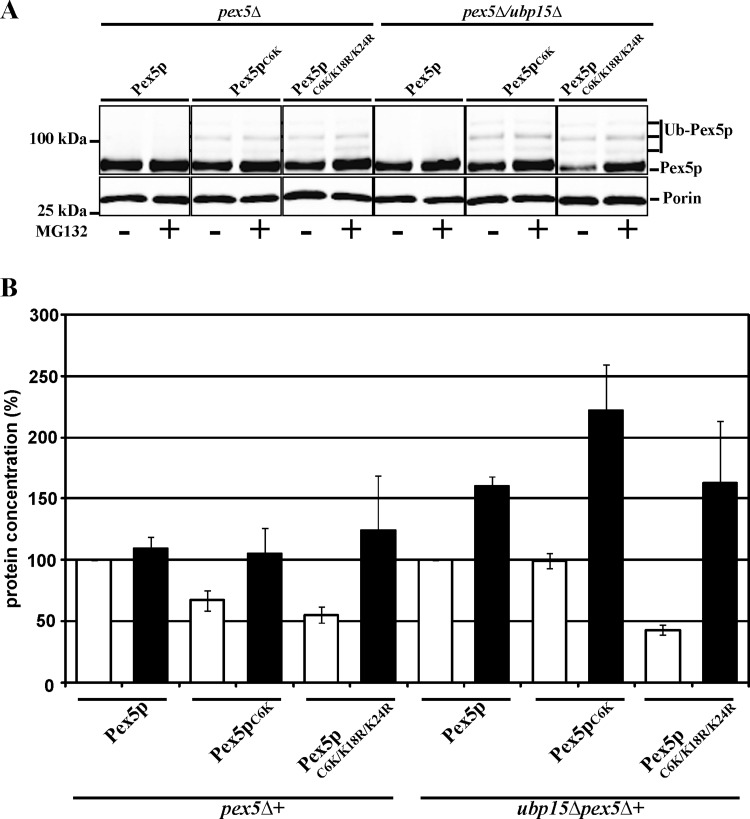
Rapid proteasomal degradation of Pex5p_C6K_ variants Mutant *pex5*Δ cells or *ubp15*Δ*pex5*Δ cells were transformed with plasmids expressing Pex5p or indicated variants. Strains were grown on oleic acid containing medium for 16 h. Subsequently cells where shifted to oleic acid containing medium with or without 40 μm MG132 to inhibit the proteasome and grown for additional 4 h. (**A**) Whole-cell lysates of the strains were prepared and blotted for the presence of Pex5p and mitochondrial porin which served as loading control. (**B**) Pex5p signal intensities of blots depicted in (A) obtained from samples without (white boxes) and with (black boxes) MG132 treatment was estimated by densitometry. Intensities were normalized to signal from plasmid-encoded wild-type Pex5p expressed in *pex5*Δ cells or ubp15Δ pex5Δ cells respectively, without MG132 treatment. In contrast with untreated cells, Pex5p_C6K_ and Pex5p_C6K/K18R/K24R_ remained stable when the proteasome was inhibited, indicating that the observed reduced steady-state concentrations are due to a rapid proteasomal degradation. Error bars=S.E.M. with *n* = 3.

## DISCUSSION

In the present study, we analysed the function of the conserved monoubiquitination site of the PTS1-import receptor Pex5p in *S. cerevisiae*. In all known species, this position is occupied by a cysteine [49]. Work in yeasts and mammalian cells demonstrated that this cysteine is indeed modified with an ubiquitin-moiety via an uncommon thioester-bond [16,38,40] and that this modification is essential for the recycling of Pex5p and therefore also for peroxisomal matrix protein import in general [12,14,38].

Ubiquitination of substrates via esterification is a rare type of modification, because in most cases the ubiquitin-moiety is attached to a lysine residue via an isopeptide bond [26,50,51]. Therefore, the functional relevance of the ubiquitin esterification of the PTS1-receptor is a central question for the understanding of peroxisomal matrix protein import. Recently, indications for a redox-based regulation of Pex5p in mammalian as well as *Pichia pastoris* cells were reported, suggesting that the conserved cysteine of Pex5p might function as redox-sensitive module [52,53]. The *P. pastoris* study suggests that the cysteine may contribute to formation of disulfide-bonds of homodimeric, cargo-loaded Pex5p molecules, which are supposed to be disassembled in the reducing condition of the peroxisomal matrix [52]. In slight contrast, the mammalian study suggests that the ubiquitination-competence of the cysteine is influenced by the redox-state of the cytosol, because oxidized glutathione interfered with monoubiquitination of human Pex5p [53]. An import-competent Pex5p-version harbouring a lysine instead of a cysteine was used as a control, as it can still be monoubiquitinated during oxidized conditions in an *in vitro* system [53]. However, the present study focused on redox-dependent effects of the wild-type human Pex5p, whereas the detailed functional consequences of the cysteine-to-lysine-mutant were not further addressed *in vivo*.

In our study, we analysed the role of the conserved mono-ubiquitination site of *S. cerevisiae* Pex5p. It has been shown earlier that the exchange of the conserved cysteine against a residue that cannot be ubiquitinated, like arginine [16], inhibits matrix protein import, blocks monoubiquitination and induces proteasomal degradation of Pex5p via polyubiquitination at the conserved lysine residues 18 and 24 [16]. This is also the case for the Pex5p_C6A_ that we have used here. Beyond this, we have exchanged the cysteine against a residue that can still be ubiquitinated, namely a lysine. Substitution of the conserved cysteine residue of mammalian Pex5p by a lysine did result in a quite functional protein [54]. Also, the yeast Pex5p_C6K_ mutant was still able to complement a *pex5*Δ strain and to facilitate matrix protein import. This finding demonstrates that esterification of the Cys^6^ is not essential for the function of Pex5p under standard conditions, as long as this position harbours a lysine as target for ubiquitination.

Interestingly, we found that Pex5p_C6K_ was polyubiquitinated. Until now, polyubiquitination of Pex5p has been regarded as part of a quality-control system that is activated when the receptor cycle is impaired. This occurs when constituents of the AAA-complex or the Pex4p-complex are mutated or when the Cys^6^ as an acceptor site for monoubiquitination is exchanged against an arginine or alanine [12,16,18,19]. With the discussed concepts in mind that the conserved cysteine might be crucial for a possible redox-regulation of Pex5p and PTS1-protein import [52,53], the polyubiquitination of Pex5p_C6K_ observed by us might simply indicate a compromised functionality of Pex5p without its supposed redox-sensor. However, our data also demonstrate that the detected polyubiquitination of Pex5p_C6K_ differs significantly from the one observed in the regular quality-control pathway, because it occurs independently of the Lys^18^ and Lys^24^. In marked contrast, the Lys^6^ itself is modified, which results in a polyubiquitin chain pattern that is distinct from the one found in a *pex4*Δ strain. The shift of the Ub-pattern intensity detected for the Pex5p of a *pex4*Δ strain to the pattern of the Pex5p_C6K_ mutant by one ubiquitin-moiety suggests that Pex4p contributes to formation of the polyubiquitin chain, possibly by attaching the first ubiquitin to Lys^6^, as it would have done in the presence of Cys^6^. However, it did not escape our attention that the Pex4p-dependent ubiquitination pattern exhibits a slight shift of the second band to a lower molecular mass (Figure 4). Our data demonstrate that the formation of an isopeptide bond instead of a thioester bond to ubiquitin, does not inhibit the function of the PTS1-receptor, but causes an unusual polyubiquitination that might also involve Pex4p. Currently, we do not know whether the polyubiquitinated Lys^6^ indeed can substitute for the function of the monoubiquitinated Cys^6^-form. It could well be that an intermediate monoubiquitinated Lys^6^ performs a so far unknown function prior to its further ubiquitination. The reason for the presence of a cysteine at this position in all species might thus not be the direction of Pex5p to quality-control and degradation. Another possible explanation considers the slightly different ubiquitination/deubiquitination rates of the thioester-linked compared with the isopeptide-linked ubiquitin. Upon Lys^6^ polyubiquitination the first ubiquitin might be bound too strong via the isopeptide bond and may stay too long at this position. This may result in further ubiquitination and formation of a polyubiquitin chain at Lys^6^ followed by receptor degradation. In general, the dynamics underlying the receptor cycle of Pex5p seem to be of importance because the import and export rates of Pex5p have to be balanced [55]. The peroxisomal membrane complexes display a limited capacity to bind PTS1-receptor molecules because a block of the export steps results in an accumulation of Pex5p at the membrane and an inhibition of matrix protein import [18–20]. Furthermore, the expression level and functional activity of docking factors and exportomer proteins have to be adjusted [56]. Moreover, both processes seem to be co-operatively coupled [57,58]. Therefore, it is reasonable that also the ubiquitination-event itself is tightly regulated. In the case of *cis*-acting factors, recent work has demonstrated that the efficient attachment of the ubiquitin-moiety to the cysteine of Pex5p and therefore functional matrix protein import requires a certain threshold of Pex4p activity, which is dependent on the C-terminal domain of Pex22p as co-activator of Pex4p [59,60]. Therefore, it seems reasonable that the retention time, which the ubiquitin-moiety stays on Pex5p, has to be limited and that the *trans*-acting factors required for the deubiquitination are tightly regulated as well. First indications that the lability of the thioester-bond might indeed be utilized for rapid deubiquitination of Pex5p were indicated by the finding that the ubiquitin-moiety could be removed non-enzymatically by cytosolic glutathione in a mammalian *in vitro* system [54]. Our data in *S. cerevisiae* demonstrate that the stronger isopeptide-bond results in an artificial polyubiquitination at Lys^6^ and therefore support the notion that deubiquitination of Pex5p has to occur relatively fast.

In line with this assumption, we were able to uncover another layer of regulation. The steady-state level of Pex5p_C6K_ is significantly reduced, when in addition the deubiquitinating enzyme (DUB) Ubp15p is deleted. Ubp15p has been identified earlier as a binding partner of the AAA-ATPase Pex6p and was shown to deubiquitinate mono- as well as polyubiquitinated Pex5p *in vitro* [30]. A functional counterpart in mammalian cells is USP9X, which also deubiquitinates the monoubiquitinated PTS1-receptor *in vitro* [48]. However, the relevance was not clear in both cases, because both enzymes seem to be partially replaceable by other ubiquitin hydrolases. In the present study, we clearly show that the combination of the *ubp15*Δ strain with the thioester-bond-replaced Pex5p_C6K_ results in a significantly reduced level of this version of the PTS1-receptor via proteasomal degradation. Taken together, these data demonstrate that under normal conditions the labile thioester-bond to ubiquitin combined with the presence of deubiquitinating enzymes like Ubp15p prevents the PTS1-receptor from being degraded and thus allows efficient recycling. These two layers of protection are quite unique. Ubp15p and its mammalian counterpart USP9X are the only ubiquitin-hydrolases that have been reported to cleave thioester-bonds of a modified substrate [30,48]. Moreover, the reason, why a cysteine of Pex5p and not a lysine is the physiological important ubiquitination site, seems to be in marked contrast with the few other known examples of non-lysine ubiquitination. This non-canonical ubiquitination of internal residues can involve the formation of oxyester-bonds to serine and threonine in addition to the thioester-bonds to cysteines [26,50,51]. Important cellular examples are the rapid degradation of the lysine-less apoptosis-supporting tBid-N after polyubiquitination on cysteine, serine and threonine residues [61]. Also the transcription factor Neurogenin 2 involved in neuronal differentiation [62,63] as well as non-secreted NS-1 (nonstructural-1 protein) Ig light chain [64] are polyubiquitinated on lysines, cysteines, serines and threonines, resulting in a rapid degradation by the proteasome. Other examples are linked to viral infections. The HIV protein Vpu (viral protein U) interacts with a cellular SCF-type (Skp1, Cullin, F-Box protein) ligase in order to promote ubiquitnation of BST-2 (bone marrow stromal antigen 2), a regulator of immunity, on cysteines, serines and threonines [65]. Moreover, the cytoplasmic tail of MHC-I in the endoplasmic reticulum (ER) is ubiquitinated on lysine, serine and threonine residues by mK3 (mouse γ-herpes virus ligase), which results in proteasomal degradation via the ER-associated degradation (ERAD) pathway [66,67]. At the cell surface, MHC-I can be ubiquitinated on lysines and cysteines by MIR1 (modulator of immune responses) and MIR2 (Kaposi's sarcoma-associated herpes virus ligases), which results in lysosomal degradation [68,69]. A closer investigation of the specificity of the RING-domain of mK3 suggested that no E2 partner specialized only on thioester-bonds is required [70]. Moreover, not the type of amino acid itself, but the position within the substrate is of importance for the ligases MIR1 and MIR2. Both can ubiquitinate cysteines as well as lysines as long as they are at a specific position [68]. This mechanistic aspect is comparable with the situation of Pex5p and the mutants Pex5p_C6A_ and Pex5p_C6A/K18C_, where a shift of the cysteine to position 18 cannot compensate the loss of the ubiquitination site at position 6. Only the exchange to a lysine at position 6 can compensate for the loss of the cysteine, indicating that the peroxisomal RING-ligase complex as well as Pex4p are not selective for the type of ubiquitinable amino acid, but for its position.

At the end, the major difference in the physiological outcome of the esterification of Pex5p at Cys^6^ to all other known examples of non-lysine ubiquitination of internal residues is that it is not intended for the degradation of Pex5p. Our data show that this modification contributes to the stability of Pex5p, whereas all other examples aim to enhance the degradation of the substrates.

Further work will analyse if a similar regulatory context exists for the PTS2-dependent matrix protein import pathway, as the PTS2 co-receptors Pex18p [24,71] and Pex20p [25,72] are also monoubiquitinated on a conserved cysteine. This would further support the novel role of ubiquitin-esterification, namely that the labile thioester-bond together with a DUB function to limit the resident time of ubiquitin on a substrate in order to prevent its degradation.

## Abbreviations

AAA: ATPases associated with various cellular activities
DsRed: *Discosoma* sp. RFP
DUB: deubiquitinating enzyme
ER: endoplasmic reticulum
MHC-I: major histocompatibility complex I heavy chain
mK3: mouse γ-herpes virus ligase
Pex: Peroxin
PTS: peroxisomal targeting sequence
RING: really interesting new gene
TPR: tetratricopeptide repeat
Ub: ubiquitin

## References

[1] Smith J.J., Aitchison J.D. (2013). Peroxisomes take shape. Nat. Rev..

[2] Nagotu S., Kalel V.C., Erdmann R., Platta H.W. (2012). Molecular basis of peroxisomal biogenesis disorders caused by defects in peroxisomal matrix protein import. Biochim. Biophys. Acta.

[3] Poll-The B.T., Gärtner J. (2012). Clinical diagnosis, biochemical findings and MRI spectrum of peroxisomal disorders. Biochim. Biophys. Acta.

[4] Waterham H.R., Ebberink M.S. (2012). Genetics and molecular basis of human peroxisome biogenesis disorders. Biochim. Biophys. Acta.

[5] Girzalsky W., Platta H.W., Erdmann R. (2009). Protein transport across the peroxisomal membrane. Biol. Chem..

[6] Purdue P.E., Lazarow P.B. (2001). Peroxisome biogenesis. Annu. Rev. Cell. Dev. Biol..

[7] Hasan S., Platta H.W., Erdmann R. (2013). Import of proteins into the peroxisomal matrix. Front. Physiol..

[8] Ma C., Subramani S. (2009). Peroxisome matrix and membrane protein biogenesis. IUBMB Life.

[9] Meinecke M., Cizmowski C., Schliebs W., Kruger V., Beck S., Wagner R., Erdmann R. (2010). The peroxisomal importomer constitutes a large and highly dynamic pore. Nat. Cell Biol..

[10] Platta H.W., Grunau S., Rosenkranz K., Girzalsky W., Erdmann R. (2005). Functional role of the AAA peroxins in dislocation of the cycling PTS1 receptor back to the cytosol. Nat. Cell Biol..

[11] Miyata N., Fujiki Y. (2005). Shuttling mechanism of peroxisome targeting signal type 1 receptor Pex5: ATP-independent import and ATP-dependent export. Mol. Cell. Biol..

[12] Platta H.W., El Magraoui F., Schlee D., Grunau S., Girzalsky W., Erdmann R. (2007). Ubiquitination of the peroxisomal import receptor Pex5p is required for its recycling. J. Cell Biol..

[13] Ma C., Agrawal G., Subramani S. (2011). Peroxisome assembly: matrix and membrane protein biogenesis. J. Cell Biol..

[14] Carvalho A.F., Pinto M.P., Grou C.P., Alencastre I.S., Fransen M., Sá-Miranda C., Azevedo J.E. (2007). Ubiquitination of mammalian Pex5p, the peroxisomal import receptor. J. Biol. Chem..

[15] Grou C.P., Carvalho A.F., Pinto M.P., Wiese S., Piechura H., Meyer H.E., Warscheid B., Sa-Miranda C., Azevedo J.E. (2008). Members of the E2D (UbcH5) family mediate the ubiquitination of the conserved cysteine of Pex5p, the peroxisomal import receptor. J. Biol. Chem..

[16] Williams C., van den Berg M., Sprenger R.R., Distel B. (2007). A conserved cysteine is essential for Pex4p-dependent ubiquitination of the peroxisomal import receptor Pex5p. J. Biol. Chem..

[17] Platta H.W., El Magraoui F., Bäumer B.E., Schlee D., Girzalsky W., Erdmann R. (2009). Pex2 and Pex12 function as protein-ubiquitin ligases in peroxisomal protein import. Mol. Cell. Biol..

[18] Platta H.W., Girzalsky W., Erdmann R. (2004). Ubiquitination of the peroxisomal import receptor Pex5p. Biochem. J..

[19] Kiel J.A., Emmrich K., Meyer H.E., Kunau W.H. (2005). Ubiquitination of the PTS1 receptor, Pex5p, suggests the presence of a quality control mechanism during peroxisomal matrix protein import. J. Biol. Chem..

[20] Kragt A., Voorn-Brouwer T.M., Van den Berg M., Distel B. (2005). The *Saccharomyces cerevisiae* peroxisomal import receptor Pex5p is monoubiquitinated in wild type cells. J. Biol. Chem..

[21] Leon S., Subramani S. (2007). A conserved cysteine residue of *Pichia pastoris* Pex20p is essential for its recycling from the peroxisome to the cytosol. J. Biol. Chem..

[22] Williams C., van den Berg M., Geers E., Distel B. (2008). Pex10p functions as an E(3) ligase for the Ubc4p-dependent ubiquitination of Pex5p. Biochem. Biophys. Res. Commun..

[23] El Magraoui F., Baumer B.E., Platta H.W., Baumann J.S., Girzalsky W., Erdmann R. (2012). The RING-type ubiquitin ligases Pex2p, Pex10p and Pex12p form a heteromeric complex that displays enhanced activity in an ubiquitin conjugating enzyme-selective manner. FEBS J..

[24] Hensel A., Beck S., El Magraoui F., Platta H.W., Girzalsky W., Erdmann R. (2011). Cysteine-dependent ubiquitination of Pex18p is linked to cargo translocation across the peroxisomal membrane. J. Biol. Chem..

[25] Liu X., Subramani S. (2013). Unique requirements for mono- and polyubiquitination of the peroxisomal targeting signal co-receptor, Pex20. J. Biol. Chem..

[26] Wang X., Herr R.A., Hansen T.H. (2012). Ubiquitination of Substrates by Esterification. Traffic..

[27] Erdmann R., Veenhuis M., Mertens D., Kunau W.-H. (1989). Isolation of peroxisome-deficient mutants of *Saccharomyces cerevisiae*. Proc. Natl. Acad. Sci. U.S.A..

[28] Wiebel F.F., Kunau W.-H. (1992). The PAS2 protein essential for peroxisome biogenesis is related to ubiquitin-conjugating enzymes. Nature.

[29] Girzalsky W., Rehling P., Stein K., Kipper J., Blank L., Kunau W.-H., Erdmann R. (1999). Involvement of Pex13p in Pex14p localization and peroxisomal targeting signal 2 dependent protein import into peroxisomes. J. Cell Biol..

[30] Debelyy M.O., Platta H.W., Saffian D., Hensel A., Thoms S., Meyer H.E., Warscheid B., Girzalsky W., Erdmann R. (2011). Ubp15p, an ubiquitin hydrolase associated with the peroxisomal export machinery. J. Biol. Chem..

[31] Güldener U., Heck S., Fiedler T., Beinhauer J., Hegemann J.H. (1996). A new efficient gene disruption cassette for repeated use in budding yeast. Nucleic Acids Res..

[32] Marzioch M., Erdmann R., Veenhuis M., Kunau W.-H. (1994). *PAS7* encodes a novel yeast member of the WD-40 protein family essential for import of 3-oxoacyl-CoA thiolase, a PTS2-containing protein, into peroxisomes. EMBO J..

[33] Liu C., Apodaca J., Davis L.E., H. R. (2007). Proteasome inhibition in wild-type yeast *Saccharomyces cerevisiae* cells. BioTechniques.

[34] Harlow E., Lane D. (1988). Antibodies - A Laboratory Manual.

[35] Albertini M., Rehling P., Erdmann R., Girzalsky W., Kiel J.A.K.W., Veenhuis M., Kunau W.-H. (1997). Pex14p, a peroxisomal membrane protein binding both receptors of the two PTS-dependent import pathways. Cell.

[36] Kerssen D., Hambruch E., Klaas W., Platta H.W., de Kruijff B., Erdmann R., Kunau W.H., Schliebs W. (2006). Membrane association of the cycling peroxisome import receptor Pex5p. J. Biol. Chem..

[37] Stanley W.A., Wilmanns M. (2006). Dynamic architecture of the peroxisomal import receptor Pex5p. Biochim. Biophys. Acta.

[38] Okumoto K., Misono S., Miyata N., Matsumoto Y., Mukai S., Fujiki Y. (2011). Cysteine ubiquitination of PTS1 receptor Pex5p regulates Pex5p recycling. Traffic.

[39] van der Leij I., Franse M.M., Elgersma Y., Distel B., Tabak H.F. (1993). PAS10 is a tetratricopeptide-repeat protein that is essential for the import of most matrix proteins into peroxisomes of *Saccharomyces cerevisiae*. Proc. Natl. Acad. Sci. U.S.A..

[40] Carvalho A.F., Grou C.P., Pinto M.P., Alencastre I.S., Costa-Rodrigues J., Fransen M., Sá-Miranda C., Azevedo J.E. (2007). Functional characterization of two missense mutations in Pex5p-C11S and N526K. Biochim. Biophys. Acta.

[41] Klein A.T., van Den Berg M., Bottger G., Tabak H.F., Distel B. (2002). *Saccharomyces cerevisiae* acyl-CoA oxidase follows a novel, non-PTS1, import pathway into peroxisomes that is dependent on Pex5p. J. Biol. Chem..

[42] Ellison M.J., Hochstrasser M. (1991). Epitope-tagged ubiquitin. A new probe for analyzing ubiquitin function. J. Biol. Chem..

[43] Swaminathan S., Amerik A.Y., Hochstrasser M. (1999). The Doa4 deubiquitinating enzyme is required for ubiquitin homeostasis in yeast. Mol. Biol. Cell.

[44] Haas A.L., Bright P.M. (1987). The dynamics of ubiquitin pools within cultured human lung fibroblasts. J. Biol. Chem..

[45] Kerscher O., Felberbaum R., Hochstrasser M. (2006). Modification of proteins by ubiquitin and ubiquitin-like proteins. Annu. Rev. Cell. Dev. Biol..

[46] Papa F.R., Hochstrasser M. (1993). The yeast DOA4 gene encodes a deubiquitinating enzyme related to a product of the human tre-2 oncogene. Nature.

[47] Weissman A.M. (2001). Themes and variations on ubiquitylation. Nat. Rev. Mol. Cell. Biol..

[48] Grou C.P., Francisco T., Rodrigues T.A., Freitas M.O., Pinto M.P., Carvalho A.F., Domingues P., Wood S.A., Rodriguez-Borges J.E., Sa-Miranda C. (2012). Identification of ubiquitin-specific protease 9X (USP9X) as a deubiquitinase acting on ubiquitin-peroxin 5 (PEX5) thioester conjugate. J. Biol. Chem..

[49] Kiel J.A., Veenhuis M., van der Klei I.J. (2006). PEX genes in fungal genomes: common, rare or redundant. Traffic.

[50] Kravtsova-Ivantsiv Y., Ciechanover A. (2012). Non-canonical ubiquitin-based signals for proteasomal degradation. J. Cell Sci..

[51] McDowell G.S., Philpott A. (2013). Non-canonical ubiquitylation: mechanisms and consequences. Int. J. Biochem. Cell. Biol..

[52] Ma C., Hagstrom D., Polley S.G., Subramani S. (2013). Redox-regulated cargo binding and release by the peroxisomal targeting signal receptor, Pex5. J. Biol. Chem..

[53] Apanasets O., Grou C.P., Van Veldhoven P.P., Brees C., Wang B., Nordgren M., Dodt G., Azevedo J.E., Fransen M. (2014). PEX5, the shuttling import receptor for peroxisomal matrix proteins, is a redox-sensitive protein. Traffic.

[54] Grou C.P., Carvalho A.F., Pinto M.P., Huybrechts S.J., Sa-Miranda C., Fransen M., Azevedo J.E. (2009). Properties of the ubiquitin-Pex5p thiol ester conjugate. J. Biol. Chem..

[55] Platta H.W., Hagen S., Erdmann R. (2013). The exportomer: the peroxisomal receptor export machinery. Cell. Mol. Life. Sci..

[56] Ratzel S.E., Lingard M.J., Woodward A.W., Bartel B. (2010). Reducing PEX13 expression ameliorates physiological defects of late-acting peroxin mutants. Traffic.

[57] Schliebs W., Girzalsky W., Erdmann R. (2010). Peroxisomal protein import and ERAD: variations on a common theme. Nat. Rev. Mol. Cell Biol..

[58] Brown A.I., Kim P.K., Rutenberg A.D. (2014). PEX5 and ubiquitin dynamics on mammalian peroxisome membranes. PLoS Comput. Biol..

[59] El Magraoui F., Schrotter A., Brinkmeier R., Kunst L., Mastalski T., Muller T., Marcus K., Meyer H.E., Girzalsky W., Erdmann R., Platta H.W. (2014). The cytosolic domain of Pex22p stimulates the Pex4p-dependent ubiquitination of the PTS1-receptor. PLoS One.

[60] Williams C., van den Berg M., Panjikar S., Stanley W.A., Distel B., Wilmanns M. (2011). Insights into ubiquitin-conjugating enzyme/co-activator interactions from the structure of the Pex4p:Pex22p complex. EMBO J..

[61] Tait S.W., de Vries E., Maas C., Keller A.M., D'Santos C.S., Borst J. (2007). Apoptosis induction by Bid requires unconventional ubiquitination and degradation of its N-terminal fragment. J. Cell Biol..

[62] McDowell G.S., Kucerova R., Philpott A. (2010). Non-canonical ubiquitylation of the proneural protein Ngn2 occurs in both Xenopus embryos and mammalian cells. Biochem. Biophys. Res. Commun..

[63] Vosper J.M., McDowell G.S., Hindley C.J., Fiore-Heriche C.S., Kucerova R., Horan I., Philpott A. (2009). Ubiquitylation on canonical and non-canonical sites targets the transcription factor neurogenin for ubiquitin-mediated proteolysis. J. Biol. Chem..

[64] Shimizu Y., Okuda-Shimizu Y., Hendershot L.M. (2010). Ubiquitylation of an ERAD substrate occurs on multiple types of amino acids. Mol. Cell..

[65] Tokarev A.A., Munguia J., Guatelli J.C. (2011). Serine-threonine ubiquitination mediates downregulation of BST-2/tetherin and relief of restricted virion release by HIV-1 Vpu. J. Virol..

[66] Wang X., Connors R., Harris M.R., Hansen T.H., Lybarger L. (2005). Requirements for the selective degradation of endoplasmic reticulum-resident major histocompatibility complex class I proteins by the viral immune evasion molecule mK3. J. Virol..

[67] Wang X., Herr R.A., Chua W.J., Lybarger L., Wiertz E.J., Hansen T.H. (2007). Ubiquitination of serine, threonine, or lysine residues on the cytoplasmic tail can induce ERAD of MHC-I by viral E3 ligase mK3. J. Cell Biol..

[68] Cadwell K., Coscoy L. (2008). The specificities of Kaposi's sarcoma-associated herpesvirus-encoded E3 ubiquitin ligases are determined by the positions of lysine or cysteine residues within the intracytoplasmic domains of their targets. J. Virol..

[69] Cadwell K., Coscoy L. (2005). Ubiquitination on nonlysine residues by a viral E3 ubiquitin ligase. Science.

[70] Herr R.A., Harris J., Fang S., Wang X., Hansen T.H. (2009). Role of the RING-CH domain of viral ligase mK3 in ubiquitination of non-lysine and lysine MHC I residues. Traffic.

[71] El Magraoui F., Brinkmeier R., Schrotter A., Girzalsky W., Muller T., Marcus K., Meyer H.E., Erdmann R., Platta H.W. (2013). Distinct ubiquitination cascades act on the peroxisomal targeting signal type 2 co-receptor Pex18p. Traffic.

[72] Hagstrom D., Ma C., Guha-Polley S., Subramani S. (2014). The unique degradation pathway of the PTS2 receptor, Pex7, is dependent on the PTS receptor/coreceptor, Pex5 and Pex20. Mol. Biol. Cell.

[73] Platta H.W., El Magraoui F., Schlee D., Grunau S., Girzalsky W., Erdmann R. (2007). Ubiquitination of the peroxisomal import receptor Pex5p is required for its recycling. J. Cell Biol..

[74] Ecker D.J., Khan M.I., Marsh J., Butt T.R., Crooke S.T. (1987). Chemical synthesis and expression of a cassette adapted ubiquitin gene. J. Biol. Chem..

[75] Kuravi K., Nagotu S., Krikken A.M., Sjollema K., Deckers M., Erdmann R., Veenhuis M., van der Klei I.J. (2006). Dynamin-related proteins Vps1p and Dnm1p control peroxisome abundance in *Saccharomyces cerevisiae*. J. Cell Sci..

